# Crisis Communication in Public Health Emergencies: The Limits of ‘Legal Control’ and the Risks for Harmful Outcomes in a Digital Age

**DOI:** 10.1186/s40504-018-0067-0

**Published:** 2018-02-06

**Authors:** Paul Quinn

**Affiliations:** Recht en Criminologie, Vrije Universiteit Brussel, Brussels Free University, Pleinlaan 2, 1050 Brussel, Belgium

## Abstract

Communication by public authorities during a crisis situation is an essential and indispensable part of any response to a situation that may threaten both life and property. In the online connected world possibilities for such communication have grown further, in particular with the opportunity that social media presents. As a consequence, communication strategies have become a key plank of responses to crises ranging from epidemics to terrorism to natural disaster. Such strategies involve a range of innovative practices on social media. Whilst being able to bring about positive effects, they can also bring about a range of harmful unintended side effects. This include economic harms produced by incorrect information and a range of social harms that can be fuelled by myths and rumours, worsening negative phenomena such as stigmatisation and discrimination. Given the potential for such harms, one might expect that affected or potentially affected individuals would be able to challenge such measures before courts or administrative tribunals. As this paper demonstrates however this is not the case. More often than not seemingly applicable legal approaches are unlikely to be able to engage such methods. This is often because such measures represent activities that are purely expressive in nature and therefore not capable of imposing any binding legal or corporeal changes on individuals. Whilst some forms of soft law may pose requirements for public officials involved in such activities (e.g. codes of conduct or of professional ethics), they are not likely to offer potentially harmed individuals the chance to to challenge particular communication strategies before courts or legal tribunals. The result is that public authorities largely have a free reign to communicate how they wish and do not have to have to comply with a range of requirements (e.g. relating to form and substantive) content) that would in general apply to most forms of official administrative act.

## Introduction

Communication is a critical part of any organised response to a crisis situation, whether the crisis be a natural disaster, an epidemic or a major terrorist attack.^1^ This includes epidemiological crises where public health experts are making ever more use of various online media. In communicating, public bodies will provide advice or other information that they feel is needed in order for the public and other actors to be able to respond in the best way possible, taking into account the situation at hand.^2^ Whilst being of crucial importance to any effort at crisis response, most crises also see criticism of the communication strategy employed. Such criticism may be inter alia because the information is incorrect,^3^ that the form in which it was made was not suitable or that is caused unneeded (social or economic) harms to certain individuals or groups.^4^ The possibility for such harms to be created by crisis communication gives rise to the question as to what options potentially affected individuals or groups may have in trying halt, alter or seek remedial measures. Whilst a range of literature exists concerning the potential social and economic harms that can (often unintentionally) be created from such programmes, there has been relatively little discussion of such aspects from a legal perspective.^5^

This paper aims to explore the limitations and possibilities of various legal approaches in being able to engage with such activity. The authors of this paper aim to illustrate that in reality there are very few legal approaches that may be able to impact directly upon public crisis communication, presenting few options for those individuals or groups who may feel harmed to seek redress). In particular, there appear to be very few if any options for individuals to challenge such activities before a legal or administrative tribunal. Where control does exist it tends to be in the form of the application professional codes of conduct, ethical codes of practice or weak forms of control exerted through the democratic process (i.e. through the executive and legislative branches). Whilst such forms of control may in theory allow some accountability for those public agencies involved in crisis communication the reality is that they may often be of little help to those who have been adversely affected (or may be at risk of being so) from such activities. This paper will attempt to illustrate both why traditional legal approaches may have difficulty in engaging such activities and why alternative mechanisms of ‘control’ may offer little to those who have suffered harms (or may be at risk of doing so).

Section 2 of this paper discusses the concept of public communication (of which crisis communication is one type) and contrasts it with other forms of activity that the state may be involved in. Section 3 will discuss the harms that can be produced from crisis communications and illustrate why the question of what controls and remedies exist is important. Section 4 looks towards international law for potentially applicable legal approaches, including in the areas of International Human Rights Law and International Humanitarian Law. This will include the ability of important legal principles found within both numerous international and domestic legal instruments such as a ‘right to life’, a ‘right to health’ and a ‘right of non-discrimination’. Section 5 will focus of the likely limited impact of approaches that are focused on privacy or data protection given that crisis communication may often occur without referring to specific individuals or using personal data. The lack of ability of administrative law (and its relevance) to impact acts that are often ‘purely expressive’ in nature is analysed in section 6. In contrast, section 7 looks at questions that arise concerning the application of hate speech approaches, laws that are designed to impact upon activity that is purely expressive in nature. Section 8 aims to highlight the importance of the relatively lack of engagement of such legal mechanisms by highlighting the limits of other alternative forms of control. This includes utilising influence that may be exerted through codes of ethics and professional conduct in addition to pressure that can be channelled through the ‘democratic processes’. In closing, section 9 will look at the good reasons that exist for the state being able to communicate in a manner that is largely free of restraint (particularly in times of crises). Such factors may not only go some way to explaining why there are few legal approaches that can be used to limit such practices, but also why various negative effects might occur if this was not the case.

## What is communication (including during a crisis)?

### Impossible to define in terms of form or content

The concept of ‘state communication’ or ‘expression’ (of which crisis communication is one of many forms)’ is not a term that one finds readily in legal literature (at least to this authors knowledge).^6^ It is not something for which in most jurisdictions there is a readily available legal definition. Indeed, most literature dealing with crisis communication or state communication in general is not legal in nature. Depending on the exact discipline involved or the particular context at play the focus may be on the efficacy of such activities, the form they take and, in many instances, the problems that arise therefrom.^7^ In such discussions the precise identity of the author of the communication (or his or her legal status) is not something that usually focused upon. The focus is usually rather on the content and the form of the communication in general. This is to a certain extent logical. Most of the academic and societal discussion concerning crisis communication for example is concerned with the effectiveness of a particular communication strategy in a particular context.^8^ On other occasions discussion may be focused on the negative effects created by such activities.^9^

As the author invoked in his introduction, there is enormous breadth and variation that may exist in terms of crisis communications. In discussing what ‘communication’ itself actually constitutes, it is of course important to recognise that such communication can vary enormously in both form and content. It is not possible to describe such activities in a concise or exhaustive way. In terms of the form, ‘communication’ in general could take the form of verbal address, the distribution of physical material (such as pamphlets and posters), letter or emails and ever increasingly, the use of communication on various forms on social media.^10^ More specifically with regard to crisis communication the type of form used will depend very much on the circumstances in question and the target audience.^11^ Often several forms may be used to disseminate the same message in order to reach a wider audience. It is not only the sheer variation available in terms of form that make an exhaustive disruption impossible but also the fact that given social and technological changes and evolutions, new forms of communication are becoming ever more effective. In the domain of crisis communication the use of social media forms of communication have become ever more popular, including in the context of public health crises.^12^ Such methods allow public authorities to harness the power of private individuals and organisations to further disseminate their desired message. Given the constant and incessant evolution in the nature and scope of social media a simple definition of such processes is largely impossible (even if this were not the case it would be far beyond the scope of this paper).

In terms of substantive details, the content will necessarily depend on the particular context in question. Within crisis contexts, this could for example involve the need to take particular security measures (e.g. in incidents related to disorder or terrorism),^13^ advice on where particular forms of healthcare treatment (e.g. in an epidemic)^14^ should be sought or advice on where food and shelter can be found (e.g. within the context of a a natural disaster).^15^ The question of what represents a crisis may receive a different answer in one context and culture than it would in another. The potential variety in terms of aims and forms means that it is very difficult to come up with a concise definition of what exactly constitutes communication by reference to certain forms or content.

For the purposes of this paper the author would suggest that it is useful to look at other factors that may aid in discerning what exactly state communication (including in public health emergencies) constitutes and how it can be separated from other activities of the state. The author of this paper would suggest two criteria that may be of use for this purpose (discussed in (b) and (c) below). These first is the need for such activities to have an ‘expressive component’, the second relates to their nature as activities that are neither ‘legally’ or ‘corporeally’ binding. An appreciation of such elements is important because, as the author will subsequently discuss, it helps to explain both why such activities can in certain occasions be harmful and also why various legal approaches are often unable to directly engage with them.

### Communication will have an ‘expressive component’

One obvious requirement that must be present for an activity to be considered as an instance of ‘communication’ is that it must have an ‘expressive component’, i.e. that it must have meaning. In the context of a particular public health crisis, efforts at communication are usually infused with explicit meaning related to the crisis, usually in terms of information surrounding its nature and/or advice or recommendations on action that should be taken. This may be the case for instance where the message in question instructs people to take a particular vaccine or call a certain number if symptoms appear. In such cases the meaning of the particular expression is objectively evident for all to see and is open to little or no interpretation. It is also important however to remember that messages may have implicit content and that such content may or may not be intentional. Implicit content can be perceived where the actual direct content of the message can be interpreted in a particular way to derive further meaning. Such interpretation will depend on the particular context involved, including the threat posed, the potential audience and the socio-economic, cultural or religious context in question. Imagine for instance public health messages stating that individuals from certain communities, or others who engage in certain behavioural practices should be aware of the the risks of contracting a certain condition and if necessary seek medical help (e.g. testing or treatment).^16^ Whilst it might not be stated so explicitly, such a statement could be seen as implicitly confirming that the public organisation involved believes that individuals from the concerned groups pose a greater risk than others in society.^17^ Whilst explicit meaning may be relatively easy to discern, the implicit meanings (real or perceived) behind may be more subtle. Often, implicit content may only be perceived by individuals in certain contexts who feel implicitly targeted by the message in question. In many instances such implicit content may not have been perceived by the author of the message in question but, may nonetheless have been perceived by certain addressees of the message in question.

### Communication activities do not entail ‘corporeal’ or ‘legal’ effect.

Another important manner of defining exactly what activities of communicational nature are (including in the context of a public health crises involves identifying those aspects are absent in communication but which are present in most other forms of activity. The author would submit that one possible way of differentiating expressive activities from other types of activity is that such activities, although having ‘expressive meaning’ do not have any form of ‘legal or direct corporeal’ effect. This is because the individual recipients of such messages are (at least in theory) able to ignore such communication and act how they might wish. Unlike for example a decision not to grant licence to drive, planning permission to build a house or to fund certain healthcare services (activities which also have ‘meaning’), activities that are purely expressive in nature have no such binding legal effects.^18^ This means that they are not able to alter the legal rights and duties that may be incumbent upon individuals either to society (i.e. the state) or to other private individuals and organisations. Nor do they have any direct physical (or corporeal) effects on individuals such as detention, quarantine or compulsory vaccination might have for example (i.e. methods that may often be associated with crisis events) .^19^ Whilst individuals may legally be compelled to pursue (or not permitted to be able to pursue) a particular course through a number of mechanisms and decisions of the ‘state’, activities that are purely expressive in nature (such as crisis communication) are not capable of entailing such compulsion.^20^ Individuals to whom they are directed can, after listening, reading or watching, simply act as they wish, even if this may be in a manner that is contrary to that which was proposed by the communicative activity in question.

As the author of this paper will demonstrate, it is this feature of activity that is solely expressive in nature that often makes it difficult for many legal approaches to engage them. Many such approaches (especially those that can be invoked by individual citizens in a court of law) seemingly depend upon the creation of corporeal or legal effects upon individuals to be capable of application. This inter alia makes its consequently difficult for affected individuals to challenge such measures in court using legal frameworks that, in many other instances, are able to provide assistance (i.e. instances where binding effects are imposed).

## Crisis communication can bring about serious harms, providing a need for careful governance

### The need to consider the existence and permissibility of potential harms?

Whilst communicative efforts in crises can and do bring about important positive effects they are also capable of having a range of unintended negative effects (described below). Such effects are capable of occurring even though acts of communication themselves are not capable of imposing directly on individuals themselves. Those involved in communication efforts on behalf of public agencies, (e.g. in areas connected to public health issues), are often very much aware of the potential for such harms to be generated as a result of their actions.^21^ Given the important role communication has to play in the response to crises and the potential for such communication to produce harms (especially when conducted in an improper manner), there is consequently a need to subject such efforts to scrutiny. Public officials will (hopefully) often be aware of the need to scrutinize crisis communications for the presence of elements that may cause unnecessary harm or reduce the efficacy of their efforts. It is in particular necessary to ask three questions concerning the use of communication in such instances:

- *(i)Is the information provided accurate?*

In crisis situations the provision of timely information can both save lives and reduce potential economic harms.^22^ It can allow individuals to take precautions or avoid behaviours that may put them at risk. Where incorrect information is however provided this will evidently not be the case. The use of inaccurate information in crisis communications can produce a number of negative consequences. Most important amongst these is a lost opportunity to provide assistance with regards to the crisis at hand and thus prevent harms occurring to individuals and society in general. In addition to the harms that occur to individuals, groups and their interests in society, incorrect communication also represents a waste of public resources that could be used more effectively. The emission of incorrect information will furthermore result in an erosion of trust of the public authorities concerned, reducing their ability to be effective in future crisis.^23^ As a consequence, there is an obvious need to ensure that information emitted in such instances is correct.

- *(ii)Can the information provided cause harm to certain groups in society?*

Whilst accurate information may allow individuals to protect themselves and their interests in the time of a crisis, it may also bring about negative consequences for certain individuals and groups in society. Such harms can occur both on the economic and social levels. In terms of the former this may occur for example where where individuals avoid visiting certain locations or forego certain activities because they are perceived to pose a risk.^24^ Such harms may occur both on the micro or macro levels (for example in the areas of tourism and transport)^25^ potentially causing great economic damage. Such problems have been observed in numerous instances including epidemic events involving H1N1 influenza, SARS and more recently the Ebola outbreak in West Africa.^26^ In each of these instances economic harm was caused because of incorrect information that had been disseminated by various agencies.^27^ Unfortunately for those concerned, the harms caused were capable of continuing long after incorrect information had been corrected. This is because of a ‘genie out of the bottle effect’ whereby once it has been disseminated, false information can not simply be retracted and will likely be further disseminated by private individuals and actors.

Communication by public agencies does not occur in a vacuum. It occurs in an environment where individuals and communities are continuously communicating with and between each other. The intensity and frequency of such communication has increased with the rise of social media.^28^ During crises there is often a dearth of available information from official sources, something that often increases individual receptivity to circulating rumours and myths that seemingly offer information.^29^ In such an environment private individuals (especially through social media) are able to potentially magnify their own opinions so that they can be heard by a great many individuals. In the context of a public health emergency for example such an environment provides fertile bedding for the establishment of harmful myths and rumours. The targets of such rumours are often pre-stigmatized groups that were already in positions of vulnerability (e.g. foreigners, those with alternative lifestyles/sexual orientations).^30^ It is therefore important that the communication of public authorities during such difficult periods does not facilitate the establishment or dissemination of such rumours or myths.

Whether the information that is provided is accurate or not it may lead to problems such as stigmatisation and discrimination for vulnerable groups. These are closely related phenomena yet are subtly different.^31^ The former relates to the induction of potentially harmful psychological states in individuals. The ability to stigmatise and be stigmatised is closely linked linked to our social nature in which the opinions of our peers and those we interactive with are of considerable importance. Stigmatisation occurs when individuals believe (rightly or wrongly) that others in society hold negative opinions about them because of a particular trait that they posses. Often such traits relate to membership of a social or ethnic group, engaging in particular behaviour or originating from a certain place.^32^ In the context of public health crises, all of these traits may be linked to threats that other individuals may want to avoid (such as a contagious epidemic).^33^ This may for example be because an infectious virus is thought to have originated in a particular place or because certain behaviours pursued by certain groups are perceived to come with a higher risk of infection. Stigmatising myths can be fuelled by both rumours (especially when they are not tackled) and also, as examples in previous crises have shown, official sources of information. For individuals who are stigmatised, the consequences can be dramatic. In the context of a public health crisis, stigmatized individuals may avoid treatment and testing because they fear being exposed to the judgemental attitudes of medical professionals who they believe are likely to hold negative feelings concerning them.^34^ Even after such a threat has been removed problems of stigmatisation may remain for some time afterwards. These include the threat of internalisation of the negative opinion of others and the employment of harmful coping mechanisms that may result in avoidance behaviours in other areas such as educational and employment opportunities.^35^ Such outcomes can worsen problems of ghettoization for vulnerable groups where they exist.^36^

Stigmatising environments can also fuel acts of discrimination.^37^ Discrimination in such contexts occurs where individuals are motivated to treat individuals differently than they should otherwise do so. This may for example be because they feel that such individuals pose a threat or because they bear moral responsibility (e.g. for being at risk of or contracting a contagious disease).^38^ Such beliefs may be fuelled by inaccurate information that individuals have received and also by myths and rumours that circulate in their respective community.^39^ Unfair treatment in the context of crises can mean the denial of access to important goods or services, including those that may be of critical importance (such as healthcare). As with the stigmatisation that often fuels it, problems associated with discrimination may often persist long after the immediate crisis has ended.^40^ Where this happens individuals from affected groups may be disadvantaged in various domains of life long after the crisis in question has ended.

- *(iii)Are any harmful effects proportional?*

In many instances the creation of harms such as those discussed above may be avoidable through carefully wording crisis communication (for example by not using unnecessarily stigmatising language). In other instances, the very nature of the communication that must be engaged in may be inevitably stigmatising for certain groups in society. Whilst such communication may bring about harmful effects for a particular group it may nonetheless be important to view such harms in the wider context, including the overall positive effects that can be achieved. Whilst in many instances the creation of such harms may be proportional given the goal concerned (e.g. protecting human life), in other instances it may not be. The question of whether such harms may be justified or not is a complex one that requires careful, considerate^41^ and well informed deliberation in the specific context in question. Identifying potentially harmful effects may not be a simple exercise. It may for example require a high level of interaction with potentially affected groups and a willingness to listen to their concerns.^42^

### A role for the law in ensuring good governance?

The ability of public crisis communication to bring about harmful effects (and even in certain occasions aggravate the nature of the crisis in question) raises the question of whether there should be mechanisms in place for individuals to challenge the form and content of certain communication programmes. Given the complexity and potential gravity of such phenomena, the need for governance processes that ensure such controls occur is seemingly clear. Such a need reflects and imperative to discern what harms might be capable of occurring, inter alia though an interactive discourse with potentially vulnerable minorities. Where necessary it requires that communication strategies are altered and that harmful practices are halted. From the perspective of a legal scholar (such as the author of this paper) one might ask what role legal approaches can play in such a governance role? Furthermore, one might arguably expect that legal mechanisms should exist for individuals or groups that by may be effected by such communication programmes to scrutinize for or enforce correct governance, or where necessary to intervene or possibly allow remedial action where harm has been caused. The following sections of this paper will argue that such a possibility may often be lacking by making references to a number of legal approaches that one might expect to be applicable in such instances.

## Crisis communication in international law

International law is the law applying to and between sovereign states. It determines their rights and obligations to each other and sometimes to third (non-state) parties.^43^ Its can be created through treaties between states or arise through a practice that has become generalised across both time and geography (i.e. customary law).^44^ The application of rules of international law to crisis communications of the state is not always evident. There is for example little specific mention of obligations in the area of ‘crisis communication’ in international law. One must rather look at requirements for example relating to the management of crises in general that may be capable of applying implicitly to the communicational efforts that they involve. Other potentially applicable categories can be found in both International Humanitarian Law and International Human Rights Law.^45^ Although most do not apply primarily to crisis situations they provide for more general human rights that could also be considered as being applicable in such situations. Some of these are discussed below. Despite their variation, they all have two common properties relevant to this paper. First they do not apply specifically to matters of communication (in a crisis or otherwise). Second they offer little or no possibility for individuals who feel that their interests have been harmed to seek redress in a court or other tribunal. In considering the practical relevance of particular instrument it is necessary to take both aspects into account. This last aspect in particular often means that the practical relevance of instruments that may seemingly be relevant on paper may be limited in reality, especially during moments of crisis where the importance of non binding international agreements is not likely to be at the forefront of a public agency’s thinking.

### Relevant elements of international law for crisis communication

In 2005 the World Health Organisation (the WHO), through its World Health Assembly released a revised version of the International Health Regulations (the IHR).^46^ It represents perhaps one of the most well known international agreements concerning the response to a crisis (in this case relating to epidemics of infectious disease).The IHR has been created in order to allow the international community to respond in a collective manner to public health threats. Amongst these changes a new and much more potent surveillance systems was introduced.^47^ It contains a range of provisions that are applicable to responses to such a crisis, including a requirement that such a response be respectful of “human rights and dignity”.^48^

Upon analysis however it becomes quickly apparent however that the IHR’s reference to Human rights is very general and does not provide specific guidance on individual rights or how they should be protected in practice. Nor does it describe specific obligations in terms of communication activities. It does however oblige states to be prepared for an epidemic situation and it also obliges “respect for human rights” in such planning. One can say that states have an obligation to consider’ human rights implications during planning for a potential epidemic situation under their obligations under the IHR. Where communication issues give rise to potential problems in terms of human rights the IHR could therefore be thought to have some implicit bearing.

The UN General Assembly has enacted resolutions calling upon states to take undertake certain responses in crisis situations. This includes UN General Assembly Resolution 46/182 for example. This resolution includes a number of obligations that may be relevant to crisis communication. This includes Article 4 which states that "Each State has the responsibility first and foremost to take care of the victims of natural disasters and other emergencies occurring on its territory…" Article 20 states " Early-warning information should be made available in an unrestricted and timely manner to all interested governments and concerned authorities, in particular of affected or disaster-prone countries…".

Perhaps the most relevant international agreement to this paper is the Hyogo Framework.^49^ It makes similar calls for preparedness in crisis situations.^50^ It defines its primary aim as "The substantial reduction of disaster losses, in lives and in the social, economic and environmental assets of communities and countries.".^51^ Interestingly it recognizes that disasters give rise to important "physical, social, economic and environmental vulnerabilities.' It furthermore states that "cultural diversity, age, and vulnerable groups should be taken into account when planning for disaster risk reduction, as appropriate.”^52^ Such obligations appears to require states to be aware of problems such as stigmatization and discrimination that may be suffered by vulnerable groups in many disaster contexts. It also states that “both communities and local authorities should be empowered to manage and reduce disaster risk by having access to the necessary information, resources and authority to implement actions for disaster risk reduction". In terms of communication the framework demands that states: "Develop early warning systems that are people centered, in particular systems whose warnings are timely and understandable to those at risk, which take into account the demographic, gender, cultural and livelihood characteristics of the target audiences, including guidance on how to act upon warnings…”.^53^ Again such an obligation seems to imply the need to plan a for a system of disaster communication that will take into account inter alia the particular vulnerabilities that a number of groups in society might face.^54^

It is important to remember however that none of the agreements or resolutions discussed here are associated with any meaningful enforcement mechanism, let alone any possibility for individuals that feel they have been negatively affected to take action. This means that whilst they may be of use in focusing political pressure upon governments they are not likely to be of direct relevance in the context of a crisis itself.

### International human rights law

There are a number of treaties that have given rise to the concept of ‘International Human Rights Law’. Each has the aim of providing private individuals with rights *vis-á-*viz sovereign states. Some of the most prominent example of such instruments on a global level are the Universal Declaration of Human Rights, the International Covenant on Civil and Political Rights (ICCPR),^55^ the International Covenant on Economic and Social Rights (ICESR) and the International Convention on the Elimination of Discrimination (ICEERD). At the the regional level important conventions such as the European Convention on Human Rights (ECHR) and the Inter American Convention on Human Rights (IACHR) may have a role to play.^56^ The ability of human rights systems to help balance competing claims by various rights holders upon limited resources arguably makes them suitable in assessing a state’s response to a crisis. States are obliged through both international law and their own national law to respect a variety of human rights principles. Important principles to consider are inter alia the right to life, the right to health, freedom from inhuman or degrading treatment, rights of physical freedom, the right to a private and family life and also freedoms against discrimination. These principles have been selected because of their prominence not only in many international legal instruments but also within national legal systems. The potential relevance of such rights to crisis communication is discussed below. Once again, as the sections below indicate the potential importance of a particular principle is not only dependent upon its seeming relevance in an abstract sense, but also the availability (or not) of any enforcement mechanisms that may be associated with it.

#### A right to life

The ‘Right to Life’ is one the cornerstone civil and political rights. It is most often concerned with negative duties upon states (i.e. the state being bound to avoid taking the life of individuals unnecessarily). The right to life though has, with the progression of time, also become associated with some positive duties incumbent upon states. The UN Commissioner for Human Rights has for instance criticised interpretations of The Right of Life that are too narrow in scope stating that “it would be desirable for state parties to take all possible measures to reduce infant mortality and to increase life expectancy, especially in adopting measures to eliminate malnutrition and epidemics”.^57^ This represents a very wide interpretation of the ‘right to life’ that is by no means universally shared. Whilst there have been cases in some states where a very expansive definition has been used, these cases are not the norm.^58^ In Europe^59^ the ECHR represents a more restrictive view of the right to life with regards to healthcare and related practices. Most cases are concerned with issues such as the right to access abortion or the duty of the state to contain or restrict dangerous substances^60^ or other hazards.^61^ The right to life is usually invoked in cases where there is a clear and relatively easily remedied threat to human life. Under most frameworks (including the ECHR) the right to life has not been interpreted as compelling public authorities to make decisions in one way or another concerning the allocation of funding to particular healthcare expenditure, even where such decisions may have involved increased risks of mortality for certain groups suffering from certain conditions. Given these factors the possibility for individual claimants to make use of this right in situations where they feel aggrieved or harmed by certain communication policies appears limited, especially given that it may be difficult to demonstrate a threat to life from such practices. This will in particular be the case where the harm in question may relate to stigmatisation and discrimination or material or economic harms. In such cases link may be far from evident.

#### A right to health

A positive duty to provide healthcare (and related services) finds for itself a more natural home within the right to health, located in various international treaties. Article 25 of the Universal Declaration of Human Rights for example outlines a ‘right to health’. This right was further clarified in article 12 or the ICESCR which recognises “the right of everyone to the enjoyment of the highest attainable standard of physical and mental health.” At the European level, article 11 of the European Social Charter^62^ for example obliges state signatories to take appropriate measures designed to prevent as far as possible epidemic and endemic diseases.^63^ A ‘right to health ‘appears to have an obvious relevance to crises that involve threats to human health, e.g. epidemic events or natural disasters. Such rights may be of little use however to those that feel aggrieved because of communication programmes for three main reasons.

The first is that a ‘right to health’ obviously does not imply that states are under an obligation to ensure that all of its citizens are in good health as such an undertaking would not be feasible. The requirements under this article have been described as representing the healthcare duties of compassionate societies towards its individuals.^64^ The Committee on Economic and Social Rights (CESR) has clarified that there is a duty upon states in terms of a right by individuals to access services and goods needed to maintain the highest possible standard of health.^65^ Under such circumstances a right to health is more likely to translate itself into a duty to make real and concrete efforts in time to acquire or produce more of the treatment in question so as to be able to treat the rest of the population as necessary.^66^ This for example is what is envisaged in many plans for dealing with a future unknown outbreak of infectious disease.^67^ It does not therefore seem likely, given this perceived duty of ‘progressive realisation’, that states will have to directly modify their immediate responses to epidemic outbreaks of infectious disease, including the communicational elements of such plans.

Second, it is also important to acknowledge the legal context of such rights and its relevance in terms of the ability of individuals who claim to have suffered harms to seek effective redress before a court or tribunal. Whilst this right may well be found in international treaties such as the ECSCR and the UDHR, these agreements are not known for providing firm options for effected individuals to challenge activities that may have negatively effected them.^68^ In contrast, a ‘right to health’ is not found within the ECHR – a human rights instrument that is associated with stronger options for appeal and redress (and which is incorporated into the legal systems of many of the member states of the Council of Europe).

Finally as with the discussion concerning a right to life above, it is important to acknowledge that that even if a right to health could be seen as giving rise to concrete requirements concerning communications in crises such as epidemics it might be difficult to make a link with practices that are for example ‘merely stigmatising’. The relationship between phenomena such as stigmatisation and human health is complex.^69^ Whilst there are certainly links between stigmatising situations and harms to human health, they may not always be apparent and may be difficult to demonstrate in reality. As section 3 discussed, stigmatisation may arise as an unintended effect caused by communication that may have an important overall positive effect. Given that such effects may relate to important goals such as protecting human life it may therefore be difficult in many cases to argue that such harms (e.g. stigmatisation or an increased risk of discrimination) would not be proportionate.

#### A right not to be discriminated against

In each of the international and regional human rights instruments discussed above the principles of non-discrimination and equal protection irrespective of race, ethnicity, social or other status are enshrined. Important examples include article 7 of the UHDR and article 14 of the ECHR.^70^ Such laws also exist at the national level in many states.

Anti-discrimination approaches are important in preventing the unfair treatment of individuals because of a certain property they are thought to possess.^71^ They have been particularly useful in terms of access to employment, education and various other public services where individuals have, for one reason or another been denied something because of a particular property they possess (or are thought to possess). Within crisis situations such laws prevent certain vulnerable categories of individuals from being unfairly treated based on factors that should not be considered. Acts of discrimination can be an important source of stigmatisation for vulnerable groups and minorities, including in crisis situations.^72^ Whilst the role of anti-discrimination approaches in general is therefore important, their ability to directly engage communication strategies themselves may often be limited. This is primarily for three different reasons (which the author has discussed in a recent paper).^73^

First, and perhaps most importantly, is that many anti discrimination approaches apply in instances where unfair ‘treatment’ results in binding legal changes or some physical imposition upon certain individuals, i.e. they impose a form of ‘treatment’. This may occur for instance where public gatherings are prohibited, where licences are not granted or where access to public services are restricted. In such activities a common element is the imposition of binding legal changes on particular individuals. Such impositions however are not directly produced by activities that are purely expressive in nature such as communication programmes. This is because whilst expressive activity can be persuasive and suggestive, individuals are not bound by it. They can (at least in theory) ignore it and act as they would whish.^74^ Given this, many anti-discrimination approaches are not likely to be able to engage purely expressive acts such as crisis communication, particularly where such expressive activity is not accompanied by legally binding or corporeal measures (e.g. relating to access to healthcare, quarantine or other detention measures). This is particularly the case with general anti-discrimination approaches which often foresee a central role for the concept of ‘treatment’ in deciding what constitutes discrimination (the ECHR seemingly being a prominent example). The practical result of this situation is that it is unlikely that individuals who feel stigmatised or otherwise effected by the contents of public crisis communications are unlikely to be able to challenge them in a court or law using such anti-discrimination laws.

The second reason relates to the fact that many anti-discrimination approaches are designed so as to apply only in specific contexts. This tends to be the case with anti-discrimination approaches that do not have as strong a focus on the existence of ‘treatment’. Whilst certain specialist anti-discrimination approaches may be capable of applying to certain expressive activities,^75^ (i.e. those that seemingly doe not involve ‘treatment’ as discussed above) they are unlikely to be capable of applying to expressive activities such as crisis communication. This is because such approaches only apply in restrictive contexts and often to a restrictive group of potential categories of individuals.^76^ They may for example only be capable of application within employment relationships or the provision of goods or services. The use of communications within a crisis situation such as an epidemic is unlikely to fall within such a context.

Third – it is important to remember that many anti-discrimination approaches apply only to acts of discrimination made with respect of certain categories (e.g. based on ethnicity/religion/sexuality).^77^ These categories are often exhaustively defined and often based upon prominent minority groups that have been known to be be historic victims of discrimination. As some of the examples discussed earlier in this paper have shown, the stigmatisation that individuals may suffer in times of crisis communication may be difficult to fit within such a category. e.g. because of their health status or the existence of a certain lifestyle or behaviour. In many instances it may be difficult to apply the common categories found in anti-discrimination legislation to the complex harms that are brought about inter alia by stigmatizing public communications activities (imagine for instance communication that stigmatises the homeless, drug users or those that engage in alternative forms of lifestyle).

## Privacy Laws

Privacy approaches can inter alia be used by individuals to prevent public bodies from using their information without their consent (or where consent is not required to restrict what can be done with such information).^78^ Data protection approaches (that apply where data that can be linked to a specific individual is used) are for example becoming increasingly common and often play an important role in governing the use of such data, particularly where the data in question is of a sensitive nature.^79^ Data protection approaches, including the EU’s data protection framework, are an important example of such approaches.

This may be case for example with regards to data relating to health or issues such as criminal convictions. Such approaches aim to protect informational privacy by inter alia providing individuals with autonomy and transparency over how their information is used for example by requiring informed consent for the use of health data.^80^ In addition they can create important conditions that must be met for the processing of data.

Whilst such legal provisions may be of application where personal data is used, they may be of little use where communication practices do not make use of personal data.^81^ This may often be the case for example where public health communication in the context of an epidemic or natural disaster is concerned. In such contexts the information distributed usually does not relate to specifically identifiable individuals. In other instances it may relate to general risk factors that may exist (e.g. terrorism) or measures that should be taken to minimalize risk. It may for example call on people with certain lifestyles to be aware of risks that they are exposed to or to seek testing but will usually not make reference to specific people. Whilst (as section 3 discussed) such communication can nonetheless be stigmatising, it can be so without referring to specific individuals. Simply invoking links between certain groups, places, or behaviours and negative connotations (such as a higher risk of contracting illness) can be stigmatising for many groups in society and therefore be responsible for a range of negative effects. The lack of any information relating to a specific individual will likely mean that many available privacy approaches will be be of potential application. This is for instance confirmed in the EU’s recent ‘General Data Protection Regulation’ which states that "the principles of data protection should therefore not apply to anonymous information, namely information which does not relate to an identified or identifiable natural person…".^82^

Similar issues may also exist when considering the application of other legal approaches related to informational privacy which often also require the utilisation of information relating to specific individuals in order to be applicable.^83^ Article 8 of the ECHR is a prominent example. The European Court of Human Rights has, in its case law, has used this article to develop a range of requirements intended to protect individual privacy. Whilst the court has interpreted the concept of privacy in a wide manner, there is no case law to suggest that it would see the use of generalised information (i.e. not relating to any specific individual) in communicational activities as bring capable of violating article 8. Thus far, the court has only found an engagement of Article 8 concerning privacy issues that involved the use of information that could be directly linked to specific individuals. Once again therefore it is unlikely to envisage individuals being able to use such case law to challenge generalised messages that do not make mention of specific individuals.

## Administrative *Law*

### Administrative law as a tool for those affected by incorrect decisions made by public officials

Administrative law is often the first port of call for individuals who feel that they have been negatively affected by incorrect decisions made by public officials. Defining administrative law itself and its functions is no easy task. It takes differing forms and is based on differing philosophies from state to state. Many civil law systems such as France for instance foresaw a unique system of law that would regulate the conduct of the state, whilst in Britain (and other English) speaking countries the prevailing philosophy demanded that the state be subject to the same system of law as private citizens.^84^ Despite these differences, one can see that to a large extent they often perform a similar function.^85^ Each can be said to be involved in the regulation of the many and various ways a state is capable of interacting with private individuals (both natural and legal) in exercising its public functions.^86^ In performing this function the aim of administrative law is to curb the excesses of government power, ensuring that it is utilized in accordance with the laws that have empowered it and often to ensure that decisions are made in line with recognized norms associated with good practice. Such systems add to the accountable nature of the state by providing extra means of control, means that are able to complement those provided by suffrage and the possibility it brings of democratically choosing the government in question.^87^ The existence of administrative law frameworks are needed to counterbalance the risks that public officials involved might exercise their discretion in an improper manner.^88^ Given the purpose of such legal frameworks it is logical to discuss their potential application to the communicational activities undertaken by public bodies, including in times of crisis. This is because administrative law offers the ability for individuals that may be affected to scrutinize the decisions of public officials in a number of important ways that would seemingly have relevance to communicational activities and the harms they can produce. These notably include:

- *(i)Checks on legality*

Ministers, civil servants and others that represent the state should have a legal basis for their actions. Such a basis can often exist in statutes created by the legislature and various forms of secondary legislative instruments. Such judicial checks form the oldest and most established grounds for judicial review, especially in common law countries. In the US, such grounds for review are associated with the longstanding and fundamental principal of limiting the role of government.^89^

- *(ii)Checks of form*

In addition to the concept of legality, administrative law also often requires that decisions are made in a certain manner, or in an appropriate form.^90^ Such grounds do not seek to specify exactly what course of action an administrator should choose, but pertain more to how the decision is made.^91^ At the heart of such scrutiny is the need to ensure that principles of good governance are observed. As Harlow states: "good government in the administrative context involves ideals of “openness, fairness, participation, accountability consistency [and] rationality”.^92^ Aims such as these are remarkably similar to themes of ‘good practice’ that have often been described in various forms of crisis communication.^93^

Most notably administrators are compelled to consider relevant criteria, to listen to the opinions of those who might be affected (including to those who may possess useful information unbeknown to the administrator) and to review matters for changes in circumstance.^94^ A duty upon administrators to provide reasons is particularly important in allowing individuals to understand both the motivations behind a decision and the factors considered in making such a decision.^95^ Given that such practices have been associated with reducing harmful occurrences in several forms of crisis communication,^96^ the application of administrative law in such circumstances would provide an important manner for individuals who might be concerned to ensure that they had been implemented.

- *(iii)Checks of adherence to important substantive principals*

In addition to matters of simple legality and form, an administrative or judicial review can allow decisions or actions to be assessed for their compatibility with important constitutional or other legal principals that pertain to the decision in a substantive sense. In other words administrative review can, in many circumstances, allow the actual decision itself to be reviewed in terms of its substantive content.

The particular legal principals that might apply are many and varied and depend upon the jurisdiction. They may for example emanate from constitutional provisions. These may relate to non-discrimination or non-interference with speech or the requirement of neutrality with regards to religion.^97^ On other occasions such principles may exist in specially created statutes that are designed to guide actions by the state and its officials. Such principles may even originate from international agreements such as the European Convention on Human Rights.^98^ The principles generated within such case law will often for example be taken into account in administrative hearings throughout Europe when deciding if administrative acts are acceptable.^99^ Administrative review by tribunals can therefore act as a gateway by which decisions can be reviewed in the light of such important legal principles. In essence they allow routine and everyday practical decisions to be reviewed in the light of abstract but important legal principals e.g. non-discrimination.^100^ The potential application of such principles would be interesting in the case of crisis communication given the types of harms that may be created (see section 4).

### A likely limited application to expressive acts

Whilst administrative law may therefore be important in holding public authorities to account for their actions, it may however be of little use in challenging the communication efforts of public authorities in crisis situations. Once again (as the reader will see is a recurrent theme in this paper), this will be because in most circumstances such activities do not impose binding changes upon individuals. Many adminsistrative law systems, in defining what an ‘administrative act’ is, seem to be focused on actions that create changes in the legal rights or obligations of individuals, or legally enforceable changes in particular benefits or obligations that an individual may be entitled to or held to.^101^ These concepts reflect notions of an ‘administrative act’ as an act by the state that has a discernable effect on the legal rights of individuals. Where such conditions are not present, it is likely that, in terms of administrative law at least, no administrative act has occurred.

The result of this will be that individuals will not, in most cases be able to utilize administrative law to scrutinize such processes, including before administrative courts and tribunals. The result of this may often be individuals who may be potentially effected by such efforts at communication (including during times of crisis) may find it difficult, if not impossible, to compel public officials (e.g. public health workers) to adhere to well recognized principles of good communication practices, including requirements of consultation, a duty to provide reasons and a duty to consider the harms that may be caused to various groups and individuals.

## Criminal law (hate speech)

Criminal law in the form of hate speech laws are interesting from the perspective of this paper because the intended target of such laws is usually activities of a primarily expressive nature. This means that there will often be no need for the co-existence of other corporeal activities or legally binding consequences (as is the case with some other prominent legal approaches – discussed above). Furthermore, public employees and civil servants, whilst often being shielded from noncriminal law disputes, usually do not have immunity from criminal prosecution.^102^ For this reason the potential for its application can often act (at least in theory) as a powerful disincentive to public employees considering breaking the law. This raises the question of whether such provisions (where they exist) could be used to challenge crisis communication that has caused harmful effects (for example where negative language concerning particular groups was used). In answering such a question it is necessary to look at the form most hate speech legislation takes. In the opinion of this author, there are two important factors that will in many instances limit the application of hate speech laws to crisis communications.

First, is the fact that when most observers speak of hate speech, they are usually referring to instances were expressions are targeted at particularly vulnerable minorities who have shown themselves to be vulnerable to attacks (in both the expressive and the physical sense).^103^ Accordingly, if one looks at the legislation behind most hate speech laws in most western democracies one sees that they are intended to apply only to expressions made against certain specifically defined categories.^104^ Common categories are ‘racial or ethnic identify’, ‘belief in a particular region’,’ and ‘sexual orientation’. Whilst in some jurisdictions the list may be longer,^105^ the exhaustive definition of such categories found in most legal systems means that only expressions targeted at individuals by virtue of their membership of such a category are capable of being caught by hate speech laws.^106^ In the context of harms brought about in crisis communication however such simplistic classifications may not be relevant or even applicable. Imagine for example communications that stigmatise the poor, individuals who engage in irresponsible lifestyles, the homeless, the overweight etc.^107^ Criticism of these and many other categories would not likely be seen as relating to the usual categories described in most forms of hate speech law.

The second important factor is the threshold required in terms of the expressive activity itself. Hate speech laws do not target all negative expressions aimed at such groups, but only those surpassing a certain threshold or meeting specific criteria. This may be for example expressions that can be classified as ‘stirring up hatred’,^108^ ‘inciting hatred’ or ‘inciting discrimination’.^109^ Whilst such a threshold may be capable of engaging serious expressions of hatred that threaten to bring about public disorder (in which those concerned by such expressions are likely to be victims) or where the author of the expressions directly calls for the discrimination of those concerned, it is unlikely to allow the engagement of expressions that are subtly and perhaps unintentionally negative (though yet still capable of bringing about stigmatisation and its harms).^110^ Those engaged in crisis communication and particularly those engaged in areas related to public health are unlikely to want to intentionally insult or bring about such negative consequences for groups in society. More often, their aim will be simply to reach as many individuals as possible. Where harm does occur it usually brought about in a more subtle and often unintentional manner, where efforts at communication may contain elements that give rise to stigmatisation.^111^ This means, that outside of blatant examples of intentional hate speech (which the author hopes in any event would rarely occur), the blunt instrument that many hate speech laws represent are unlikely (with good reason) to be able able to offer much to individuals who may be negatively affected by such communicative undertakings.

## Ethical codes, codes of professional conduct and the democratic process

Whilst the foregoing discussion has indicated that many traditional legal approaches (that are associated with the availability of access to courts and tribunals allowing affected individuals to seek means of redress) may have difficulty in engaging activities that are solely of an expressive nature, this does not mean however that such activities are totally free from all forms of control. Public employees involved in matters of communication are of course not simply able to say what they wish without fear of repercussion. Others forms of control exist, though they may not be ‘accessible’ to those individuals that are directly affected by the communicational activities in question (i.e. they may not for example provide access to a tribunal for affected individuals). The reality is that public employees who are involved in communicating on behalf of the state, and the organizations they work for are subject to a range controls and requirements that are able to exert influence on their discretion in terms of the content and form of the communicational activities engaged in. Such forms of control often take the form of soft law such as ‘codes of conduct’, ‘ethical codes’ and other forms of control that are linked to ‘financial exhortation’ by executives and legislatures (i.e. related to the ‘democratic process’). Some illustrative examples of such processes are described below.

### Codes of conduct

Many public employees and agencies are likely to be subject to codes of conduct. The purpose of such codes is to identify the main principles by which public employees should operate. Such codes have been becoming more common in recent years, including gaining prominence in continental Europe (where the term ‘deontological code’ is commonly use),^112^ where they have been inspired to a certain extent by the example of the UK (and other English speaking contexts).^113^ In the UK for example the ‘Civil Service Code of Conduct’ requires public employees to refrain from acting "in a way that unjustifiably favours or discriminates against particular individuals or interests". They should also carry out responsibilities in a way that is “fair, just and equitable” and reflecting a commitment to “equality and diversity”.^114^ Similar principles exist in civil services codes in many states including (but not limited to) Belgium^115^ and France.^116^ In some instances a code of conduct may exist that deals particularly with efforts at communication and may demand that civil servants communicate in ways that are not likely to worsen discrimination and create unnecessary harms.^117^

In some instances codes of conduct may exist in legislation, whilst in other instances such requirements may take the form soft law. Depending upon the the type of legal system in question the legal value of such codes may vary. In the UK, where the legal system is more open to using such forms of ‘soft law’ in ‘hard’ legal cases, such codes are likely to carry more legal weight. They may for example be understood to form part of the unemployment conditions of public employees.^118^ This raises the possibility of particular individuals being sanctioned or suffering consequences where they fail to act as expected. This could feasibly occur where they are responsible for communication practices that are contrary to whatever code a particular employee may be bound by. In continental Europe, the legal status of such codes (where they are not contained in legislation) is more dubious. Many continental systems do not recognises codes or circulars as binding sources of law. As a consequence, the contents of such codes may not bind individuals in the same manner (i.e. forming a condition of their employment). In such instances the ability of such codes to weigh on the decision making of a public employee will accordingly be reduced. Even where this is the case they may still however be used however in order to judge or appraise the behavior of public servants.^119^

### Ethical codes linked to professional status

Many individuals who act on behalf of the state in times of crisis communication may also be subject to other forms of professional codes of conduct or ethics. Unlike codes of conduct that may apply to public employees in general, such codes may apply to specific individuals by virtue of the profession they belong to. One of the most important examples of such codes are the professional codes of conduct that bind doctors, nurses and other medical professionals. In most countries such medical professionals must, in order to be active professionally, be registered with a national body, and agree to act professionally in manner that is considered compatible with their profession. Such codes aim to ensure that indiviuals belonging to certain professionals act in a way that is ethically and professionally consistent with that which might reasonably be expected. In the UK for example, doctors must be a member of the British Medical Council, which has issued guidelines on “Good Medical Practice” (including requirements related to communication and non discrimination).^120^ Where there are concerns that such standards have not been met, special panels of inquiry, described as ‘fitness to practice’ panels are able to issue warnings, impose conditions on a doctor’s practice, suspend a doctor, or even erase them from the medical register. Examples of similar bodies can be found in most other European countries, including Belgium,^121^ France,^122^ Germany,^123^ Ireland^124^ and the Netherlands.

### Control through the democratic process

In many states the judicial branch of government has an extremely limited level of control over the expenditure of various government departments.^125^ This means that a great deal of power is vested in the executive and legislative branches in terms of deciding what funding various departments receive (and by extension what activities they perform). This raises the question of whether such democratic forms of control may be of use to individuals who have been negatively affected by a particular communication programme. In reality, almost all activities (including communication) require the creation and maintenance of networks of staff and allocation of resources. Such activities are usually dependent upon decisions to provide the necessary funds and personnel available. If such resources are not allocated by the executive (i.e. by the relevant minister or one of their delegates) a communications project can not be commenced or continued. Where it is felt that a particular project would not, or is not producing the desired results it can simply be discontinued by withdrawing funding or redeploying personnel. ‘Controlling the purse strings’ represents one of the most important mechanisms of control in steering the activities of public bodies and services.^126^ Whilst such a form of control may be quick and efficient from the perspective of the executive, the ability of individual citizens or affected groups to challenge or influence such decisions is limited.

In the UK context parliament has a notional control on the allocation of funds to the executive. Such control is however in reality weak in many regards. This function is exercised by the Public Accounts Committee (the PAC), which is composed of members of parliament It is effectively limited to ensuring that the UK Treasury has received the consent of parliament for the total allocation of funds to the department in question. Such a level of control is very mild given that the department in question does not need to spell out exactly how the money it receives will be spent.^127^ This leaves the department in question with relative freedom to decide how and where its budget may be spent. Under the UK constitutional system courts have very limited powers to regulate specific allocations of departmental expenditure.^128^ This means that to a large extent, once a department has been legally allocated funds by parliament for a certain purpose e.g., public health, it is free to spend such money in any way it sees fit in pursuing aims connected to that purpose. In general the judicial branch of government may only interfere with such choices when they interfere with the rights or privileges of individuals (though as the proceeding sections of this paper have shown, activities that are merely ‘expressive’ in nature may often not be seen as doing so).^129^

In France a similar logic exists to the UK, although with more clearly demarcated institutions that are tasked with scrutinizing government expenditure. The *Cour des Comptes* (Court of Auditors) exists to examine all public accounts including those of publicly owned enterprises.^130^ Like the PAC in the UK its function is primarily that of auditor which allows it to advise both the executive and parliament as to whether departments have been spending the money allocated to them correctly. As is the case with PAC for the UK Parliament, the reports of the *Cour des Comptes* are not themselves binding, but are meant to arm the French legislature with sufficient evidence to make binding votes on public finances.^131^ It is ultimately the legislature that has the final say on the legality of allocation of state funds.^132^ To do this the *Cour des Comptes* produces reports and studies that are requested by both the *Assemblée Nationale* and the Sénat. In its scrutiny of public expenditure it has a role before the budget is approved and afterwards in an audit function. When undertaking such studies a primary aim is to ensure that money is spent in a legal way and that the spending in question achieved ‘value for money’.^133^ Where cases of illegal action are discovered the *Cour des Comptes* can invite a prosecution.^134^

### The weakness of democratic control

Given the role for legislatures in allocating funds to the executive, one might expect that private individuals might be able to influence such processes through the democratic process. This could be either through exercising one’s right to suffrage or by placing political pressure on elected representatives. The level of control that legislative bodies are able to exert on a de facto basis is however in reality weak. Legislatures may only have control in the *de jure* sense, with de facto control resting with the executive and more specifically the treasury. Legislatures do not vote on minute details of parliamentary expenditure, but approve expenditure in terms of annual budgets at the department level.^135^ Furthermore, legislatures are not likely to have the time, expertise or resources to analyze all aspects of departmental budgets in detail. Considering that some departments are very large with an enormous range of competences this is understandable.

One can take the UK’s National Health Service as a good example. It has an annual budget of 116 billion pounds which can be broken down into numerous complex smaller sub-departmental budgets including inter alia for public health information campaigns.^136^ With such large and complex budgets, parliament is unlikely to be able to analyze the spending that will be committed to individual projects (including programmes intended to provide crisis communication such as epidemics or other public health crises).^137^ Given that information campaigns and other expressive activities by the state are often opted for because they are perceived as a cheap solution,^138^ it is extremely unlikely that parliamentary scrutiny will extend to such relatively modest expenditure projects.

Other important factors adding to the weakness of such forms of control being able to prevent harms from occurring to certain groups is their ex post facto nature and their focus on primarily financial aspects. Processes of accountability (such as review by parliamentary or other institutional committees or bodies) often takes place after such spending has occurred, with the aim of reducing waste and promoting efficiency. Such reviews are primarily concerned with financial efficiency and less so with other issues such as human rights and ethics). This means that reviews of this type are unlikely to be alert to the types of harms this article is concerned with (i.e. they are not harms to the public purse). Furthermore, many of the types of harms discussed in section 4 may have already occurred by the time such a review occurs. Given the nature of harms related to phenomena such as stigmatization and discrimination, there may be very little that can be done at such a stage even if such harms were to be effectively identified.

## The pragmatic case for restrain free public communications (including in crises)

The author of this paper has thus endeavored to demonstrate that communications of the state and associated bodies and organizations face a low level of constraint, particularly when compared to many other forms of activity which public authorities may engage in. This includes communication in various forms of online epidemiology. In doing so it is necessary not only to describe why such communication may be relatively constraint fee but also pose the question as to how this may be the case. Indeed it would not be fitting to conclude this paper without at least acknowledging the positive effects that appear to exist as a consequence of the apparent lack of legal control surrounding crisis communications. This requires once again quickly reflecting on the nature of communication during a crisis and what purposes it serves.

As section 3 discussed such activities effectively represent an important category of ‘tools’ available to public authorities to respond to situations.^139^ They can arguably be contrasted with other important tools in two important regards. The first (as as been discussed at length in this paper) is that communication does not impose binding consequences on individuals: it does not compel them to do anything nor to suffer any form on obvious imposition. In short individuals can simply ignore the use of such a tool if they wish. Second, in comparison to other tools of the state, the use of communicational practices requires relatively little in terms of resources and relatively little in terms of infrastructure. They cost relatively little and do not require complex networks of personnel and materials. Furthermore, given that they do not impose upon the liberties of private individuals (either in the physical or pecuniary sense), there is often no need for a complex legal framework in place. These properties of communicational activities make them an attractive ‘tool’ that is available to the state in particular circumstances. These include incidences such as emergencies where there is a state of flux. It is precisely in such situations that a rapid and flexible response is essential. Providing individuals with timely information can be as effective as any physical measure that may be taken whilst at the same time being much less invasive. One can therefore say that the non-availability of such quick and flexible ‘informational’ measures would mean that that they state would loose a vital tool in its response to crisis situations.

Similarly, the existence of legal frameworks capable of allowing private individuals to alter or halt such processes would also represent a diminution in the utility of the communicational tools available to the state. If for example individuals were able to utilize administrative law processes to challenge particular forms of crisis communication that were employed, it could mean that public authorities were prevented from using one of their most important tools at a critical moment. Even where courts were to dismiss such interventions, the need to engage in complex legal analysis or disputes at such a critical moment would represent an undesirable distraction for public officials aiming to respond to a critical situation. By the same token, it would not be desirable for public officials to to be forced, in an unfolding crisis, to give consideration to precise and complex legal requirements concerning the form that communications must take and the content they must possess. Such a requirement would, in most instance represent a distraction from what would in most instances represent more pressing concerns.

Whilst as sections 3 and 4 discussed communicational activities, especially in the context of a crisis are no doubt capable of creating serious harms, such harms should not be viewed in isolation. This is because they are most likely to be unintentional by-products of activities that are otherwise responsible for important positive effects. When weighed against such potential benefits (including the protection of human life), the potential to create many of the types of harms discussed in this paper may seem acceptable. In addition, whatever harms that are produced by such measures are likely to be less in magnitude than other potential measures that may involve the use of physical force or the imposition of binding changes upon individuals. When compared to such potential measures, the use of expressive measures that fail to impose any concrete impositions on individuals, and which one is, in theory, simply able to ignore may be considered as representing the type pf measure with the least effect on individual liberty possible, even where they are in certain circumstances capable of brining about certain unintended effects.

## Conclusion

That crisis communication programmes can produce harms of various forms has been demonstrated in a wide variety of literature.^140^ The possibility for such harms has arguably increased greatly in the social media age. Given that there is an undoubted imperative to reduce such harms the question of what potentially affected individuals or groups can do to prevent or seek redress for such harms is important. This paper has sought to explore this question primarily from a legal perspective. In particular, it has sought to explain why affected individuals may find it difficult if not impossible to find legal approaches that can be used compel public organizations to alter or cease a particular form of communicational activity. The result is that individuals who may feel adversely affected have to rely on other weak forms of control that they have little power themselves to influence (such as forms of control exercised by the existence of codes of conduct/ethical codes or forms of control/exercised through the democratic process).

There are varying reasons why seemingly relevant legal approaches are not likely to apply to crisis communication. In terms of international law, whilst there is no instrument relating specifically to crisis communication there are instruments in both international humanitarian and international human rights law that certainly appear to be relevant. Whilst the level of direct relevance of such instruments may vary, what they have in common however is the lack of any effective enforcement mechanism for individuals that may feel that they have been harmed by crisis communication. Even where complaint mechanisms do exist, they tend to be weak in nature, essentially depending on the creation of political pressure to bring about change on the part of any state that may be concerned.

At the domestic level the picture is somewhat the converse. Whilst there is no shortage of candidate legal frameworks that are able to provide real mechanisms of redress (e.g. injunction, pecuniary compensation or even criminal conviction), affected individuals are likely to have difficulty in convincing judicial bodies that such frameworks can actually engage efforts at crisis communication. The reasons for this are the nature of such activities and the effects they are able to have on individuals and groups. In many cases communicational activities (including during a crisis) can be considered as being of a ‘purely expressive nature’. This can be contrasted with many other forms of activity that the state engages in given that such activities are not capable of having any binding ‘legal’ or ‘corporeal’ effect. The former relates to alterations in an individual’s legal order, which usually means changes in the rights and duties accorded to that individual. This could occur where for example rights to access services (e.g. healthcare) are removed or where taxes or charges are levied. It could also occur where reference to specific individuals is made or personal data is used. The latter aspect (i.e. entailing corporeal effect) relates to actions that bring about physical effects on individuals, (e.g. death, detention or a forced medical intervention).

As sections 5 – 9 showed many important legal approaches (approaches that are often of use to vulnerable groups and minorities) seemingly require the existence of such effects to be of application. This is the case both with administrative law and many anti-discrimination approaches for example. The former represents one of the most essential tools in the system of checks and balances that are designed to restrain executive action and prevent unnecessary harms from occurring. Administrative law frameworks often however require that individuals’ legal rights or duties are threatened or altered in order to be applicable. The very concept of an ‘administrative act’ for example is often formulated as a decision that has binding legal effect. The latter (i.e. anti-disrimination law) is often of application where some form of discriminatory ‘treatment’ has occurred (i.e. in the form of binding legal changes). This is often viewed as requiring the imposition of some form of binding change. Whilst exceptions may exist (the EU’s anti discrimination approaches are for example seemingly capable of applying to expressive activity), they may only apply in very specific circumstances or may only protect discrimination again certain specific categories. This means they may not be applicable to most or all forms of crisis communication. An analogous situation also seems to exist concerning the potential application of privacy approaches (e.g. data protection), where it may be necessary to show that information relating to specific individuals is being used or that such individuals have suffered harms to their privacy. Given that crisis communication does not usually refer to specific individuals but to groups or categories, this is unlikely to be the case.

A similar type of problem exists with regards to other types of legal approaches that are designed specifically to target harmful expressions (e.g. hate speech laws). Whilst such approaches do not necessarily require the demonstration of binding legal or corporeal effects, they nonetheless often have forms of conditionality that will limit their application. Many for example can only apply to expressions made against an exhaustively defined set of categorizations (e.g. race, ethnicity, religion). In addition, the threshold for the application of hate speech provisions may also be too high (e.g. the incitement of hatred or discrimination) to realistic engage the types of harm that can be produced by crisis communication. Requirements such as these may be too onerous when one is discussing the types of subtle harm that may be brought about by phenomena such as stigmatization and related harms. For such phenomena to arise, it is not necessary to incite hatred or discrimination. The existence of expressions or information that can (even unjustifiably) induce individuals to simply simply possess negative opinions concerning others (or even themselves) may be sufficient.

Whilst many traditional legal mechanisms of control may have difficulty in gaining traction with the type of purely expressive activity that communicational practices often represent, there are a number of alternative forms of regulatory control that should not be discounted. Although they are often not as accessible (and enforceable in a court of law) to individuals who may be affected by communicational activities, they may nonetheless impose an important source of restraint upon public officials that are engaged in such activities. Amongst such influences are codes of conduct, ethical codes and various rules related to professional status. Depending on the particular profession and context in question, such codes may prevent public officials from acting in certain ways that may be viewed as unethical or unprofessional, potentially including practices related to crisis communications. Where such codes are not complied with, public officials may run risks such as sanction, dismissal or even removal of professional status (e.g. doctors, nurses etc.). The binding nature of such codes, in addition to the sanctions that would be imposed in the event of breach are however extremely variable from jurisdiction to jurisdiction. In addition, the applicability of such codes to matters of communication (including during crises) may not always be readily apparent.

The final form of potential control discussed in this paper was the ability of affected individuals to exert control ‘democratically’ i.e. through the electoral process by affecting the make up of the executive and legislative branches of government. Through the appointment (or dismissal) of individuals to relevant posts or the allocation (or not) of funds to particular programmes, individuals in position of control in the executive (and often by extension the legislature) are (at least in theory) able to exercise control over what public agencies say and how they say it. The complexities of modern states and public bodies however mean that the level of control exerted by those in position of executive power over the many activities that are carried out on its behalf is not likely to be minutious. The reality is that many communicational activities that are carried out on behalf of public bodies are small in scale (especially when seen in expenditure terms) in comparison with other forms of activity. This reality means that the attention paid to particular acts of communication carried out by the executive is likely to be limited. The same principle applies to legislative control over executive activities. Whilst in principle legislatures often retain ultimate control over many types of executive activity, the ability of legislatures to scrutinize individual projects (such as for example health communication programmes) is likely to be limited. Even if such a subtle level of control was realistic, the ability of an affected individual (or group of individuals) to influence such matters through the ballot box would be extremely limited. This theoretical existence of such democratic methods therefore represents at best a poor substitute for the forms of control that binding legal approaches would be able to offer.

Whilst this paper has established that there is a lack of control concerning public communication activities in the context of crisis (and the potential issues that can arise as a result) the reader will no doubt be aware that the author has not (in this paper at least) sought to argue for an alternative arrangement whereby purely expressive acts made by the state would be subject to a much broader range of legal controls than they are at present. This is because the implications of such a change would be considerable, and are deserving of much further thought and consideration than is possible within the confines of this paper. It would also mean that the state would be deprived of perhaps its most flexible tool, i.e. the ability to say what it wants, how it wants and, in an expeditious manner in response to a crisis. Whilst this might mean that in certain occasions that individuals have the ability to challenge certain communicational practices that may have brought about negative effects for them, it would also mean that vital processes of communication that are needed to potentially protect both life and property could be subjected to burdensome legal procedures that could in many cases be capable of blunting their efficiency. Whether the types of harms that can be caused by purely expressive activities which can (in theory at least) be simply ignored would warrant such interference is a complex discussion that deserves much more attention. The answer depends to a large extent what other mechanisms for control exist. Answering this question correctly will require further research into how ‘non-legal’ methods of control operate and their ability to reduce the potential for crisis communication to cause harms (something which the author intends to pursue in a subsequent paper).

## Footnotes

1 For a good discussion on the role of crisis communication within overall disaster management strategies see: T Coombs, *Ongoing Crisis Communication: Planning, Managing, and Responding* (California: Sage, 2016). See also: A Zaremba, *Crisis Communication: Theory and Practice* (New York: Routledge, 2010).

2 T Steelman and S MCCaffrey, “Best Practices in Risk and Crisis Communication: Implications for Natural Hazards Management,” *Natural Hazards* 65 (2013).

3 C Mayhorn and A Collins McLaughlin, “Warning the World of Extreme Events: A Global Perspective on Risk Communication for Natural and Technological Disaster,” *Safety Science* 10.1016/j.ssci.2012.04.014 (2012).

4 B Person et al., “Fear and Stigma: The Epidemic within the Sars Outbreak,” *Emerging Infectious Diseases* 10, no. 2 (2004). N Guttman and C Salmon, “Guilt, Fear, Stigma and Knowledge Gaps,” *Ethical Issues in Public Health Communication* 18 (2004). G Mythen and S Walkate, “British Journal of Criminology,” *Criminology and Terrorism* 46 (2006); G Mythen, S Walkate, and F Khan, “I’m a Muslim, but I Am Not a Terrorist’: Victimisation, Risk Identities and the Performance of Safety,” *British Journal of Criminology* 49 (2009).

5 Mayhorn and Collins McLaughlin, “Warning the World of Extreme Events: A Global Perspective on Risk Communication for Natural and Technological Disaster.”

6 P Quinn and P De Hert, “Self Respect—a “Rawlsian Primary Good” Unprotected by the European Convention on Human Rights and Its Lack of a Coherent Approach to Stigmatization?,” *The International Law of Discrimination and the Law* 14 (2014). The most important exception is the concept of the ‘government speech’ under developed by the US Supreme Court under the US constitution. For more see: A Greene, “Government Speech on Unsettled Issues,” *Fordham Law Review* (2001); A Olree, “Identifying Government Speech,” *Connecticut Law Review* 42, no. 2 (2009); C Taylor, “Hate Speech and Government Speech,” *Journal of Constitutional Law* 12, no. 4 (2010).

7 See for example the description of ‘Nodaltiy’ in (chapter 2) C Hood and H Margretts, *The Tools of Government in the Digital Age*, ed. Palgrave Macmillon (2006). See also B Morgan and K Yeung, *An Introduction to Regulation* (Cambridge: Cambridge University Press, 2007). See also: M Howlett, “Government Communication as a Policy Tool: A Framework for Analysis,” *The Canadian Political Science Review* 3, no. 2 (2009).

8 See for example J Bertand et al., “Systematic Review of the Effectiveness of Mass Communication Programs to Change Hiv/Aids Related Behaviors in Developing Countries,” *Health Education Research Theory & Practice* 21, no. 4 (2006).

9 H Cho and A Salmon, “Unintended Effects of Health Communication Campaigns,” *Journal of Communicaiton* 57 (2007).

10 Discussions in both case law and academic discussion concerning the concept of ‘government speech’ as delineated by the US Supreme Court invoke such potential variation, even discussing objects such as statues. See: Olree, “Identifying Government Speech.”; H Norton and D Keats Citron, “Government Speech 2.0,” *Denver University Law Review* 87 (2010); N Murphy, “Context, Not Content: Medium-Based Press Clause Restrictions on Government Speech in the Internet Age,” *University of Denver Sports & Entertainment Law Journal* 7 (2009); Greene, “Government Speech on Unsettled Issues.” Norton for instances discussed the legal issues surrounding new methods of communication by public bodies, including the increasing use of social media.

11 The communication methods used by a crisis may for example vary at what stage the communication in question is occurring e.g. pre, during or post crisis. See: T Steelman and S McCaffery, “Best Practices in Risk and Crisis Communication: Implications for Natural Hazards Management,” *Natural Hazards* 65 (2013).

12 S Moorhead et al., n.d., “A New Dimension of Health Care:Systematic Review of the Uses, Benefits, and Limitations of Social Media for Health Communication,” *Journal of Medical Internet Research* 5, no. 4.

13 Mythen, Walkate, and Khan, “I’m a Muslim, but I Am Not a Terrorist’: Victimisation, Risk Identities and the Performance of Safety.”; K Hunt and K Rygiel, *(En) Gendering the War on Terror: War Stories and Camouflaged Politics* (New York: Ashgate Publishing, 2013).

14 Guttman and Salmon, “Guilt, Fear, Stigma and Knowledge Gaps.”

15 S Muralidharan et al., “Hope for Haiti: An Analysis of Facebook and Twitter Usage During the Earthquake Relief Efforts,” *Public Relations Review* 37, no. 2 (2011).

16 K Bell et al., “Smoking, Stigma and Tobacco ‘Denormalization’: Further Reflections on the Use of Stigma as a Public Health Tool. A Commentary on Social Science & Medicine’s Stigma, Prejudice, Discrimination and Health Special Issue (67: 3),” *Social Science & Medicine* 70, no. 6 (2010).

17 R Bayer, “Stigma and the Ethics of Public Health: Not Can We but Should We,” ibid.67 (2008). P67 The author discusses the harm that labelling can cause and how enforced labelling of various groups should be avoided where possible.

18 Hood and Margretts have given much though to the different tools of the state. They invoke the concepts of ‘nodality’ (which relates to the use of information), ‘authority’ (which relates to the use of laws), ‘treasure’ (which relates to the use of financial means and organization (which relates to the use of of physical means). In their scheme ‘nodality’ can be separated from other tools in that it does not have direct ‘effects’ on individuals but can be ignored. See: Hood and Margretts, *The Tools of Government in the Digital Age*.

19 Ibid. In their work (in chapter 5) Hood and Margretts refer to the use of such physicial asspects (including force) as the use of ‘organization’. This may for example include the use of the police to enforce decisions of the courts or other legal authorities.

20 One grey area might include order by public officials to civil servants (including the police) to take physical or legal measures. For the purposes of this paper however the author would not class such activities as communicational or expressive in nature but rather as legally binding administrative acts. As a consequence most western systems of administrative law would be able to engage such acts (discussed further in section 5)

21 J Hood and A Friedman, “Unveiling the Hidden Epidemic: A Review of Stigma Associated with Sexually Transmissible Infections,” *Sexual Health* 8, no. 2 (2011).

22 The World Health Organization has for example discussed this at length in its (2005) document “Outbreak Communication” using the SARS epidemic in 2003 as an example of how good co-ordination and communication and practice prevent an epidemic from becoming entrenched. Available at: http://www.who.int/csr/resources/publications/WHO_CDS_2005_32web.pdf See also Coombs, *Ongoing Crisis Communication: Planning, Managing, and Responding*.

23 G Böl, “Risk Communication in Times of Crisis - Pitfalls and Challenges in Ensuring Preparedness Instead of Hysterics,” *EMBO Reports* 17 (2016).

24 M Pappaioanou and M Gramer, “Lessons from Pandemic H1n1 2009 to Improve Prevention, Detection, and Response to Infl Uenza Pandemics from a One Health Perspective,” *ILAR Journal* 51, no. 3 (2010).

25 P Perry and F Donini-Lenhoff, “Stigmatization Complicates Infectious Disease Management,” *American Medical Association Journal of Ethics* 12 (2010). The authors give example of the harm that Mexican individuals and economic interests suffered around the world

26 During the Ebola outbreak false information contributed to the stigmatisation that healthcare workers and vulnerable communities felt. See: Office of the United Nations High Commissioner for Human Rights (West Africa Regional Office), “A Human Rights Perspective into the Ebola Outbreak,” (2014). Abramowitz S, McKune SL, Fallah M, Monger J, Tehoungue K, Omidian PA. The Opposite of Denial: Social Learning at the Onset of the Ebola Emergency in Liberia. J Health Commun. 2017;22(sup1):59-65. 10.1080/10810730.2016.1209599.

27 Pappaioanou and Gramer, “Lessons from Pandemic H1n1 2009 to Improve Prevention, Detection, and Response to Infl Uenza Pandemics from a One Health Perspective.” Significant economic harms were caused to swine farmers following policies implemented concerning the slaughter of pigs during the swine flue epidemic of 2009.

28 R Merchant, S Elmer, and N Lurie, “Integrating Social Media into Emergency-Preparedness Efforts,” 365, no. 4 (2011).

29 Deliverable D1.6 “Human Rights, Stigmatization and Risk of Discrimination Against Specific Population Segments and Target Groups” from the project TELL ME p23

30 Perry and Donini-Lenhoff, “Stigmatization Complicates Infectious Disease Management.”

31 Quinn and De Hert, “Self Respect—a “Rawlsian Primary Good” Unprotected by the European Convention on Human Rights and Its Lack of a Coherent Approach to Stigmatization?.”; S Burris, “Stigma, Ethics and Policy: A Commentary on Bayer’s “Stigma and the Ethics of Public Health: Not Can We but Should We”,” *Social Science & Medicine* 67 (2008); Colandrea, “On the Power of the European Court of Human Rights to Order Specific Non-Monetary Measures: Some Remarks in Light of the Assanidze, Broniowski and Sejdovic Cases,” *Human Rights Law Review* 7, no. 2 (2007); A Courtwright, “Stigmatization and Public Health Ethics,” *Bioethics* 27, no. 2 (2013).

32 A. Gluszek and J. F. Dovidio, “The Way They Speak: A Social Psychological Perspective on the Stigma of Nonnative Accents in Communication,” *Pers Soc Psychol Rev.* 14, no. 2 (2010).

33 J Williams, Gonzalez-Medina, and L Quan, “Infectious Diseases and Social Stigma,” *Infectious diseases and social stigma* 4, no. 1 (2011).

34 M Chesney, “Critical Delays in Hiv Testing and Care,” *American Behavioural Scientist* 42, no. 7 (1999).

35 J Dovidio, B Major, and J Crocker, “Stigma: Introduction and Overview,” in *The Social Psychology of Stigma*, ed. T Heatherton, et al. (New York: Guilford Press, 2000); B Link and J Phelan, “Conceptualizing Stigma,” *Annual Review of Sociology* 27 (2001); Crocker, “Social Stigma and Self-Esteem: Situational Construction of Self Worth,” *Journal of Experimental Social Psychology* 35 (1999); B Major et al., “Coping with Negative Stereotypes About Intellectutal Performance: The Role of Psychological Disengagement,” *Personality and Social Psychology Bulletin* 24, no. 1 (1998).

36 H Deacon and I Stephney, *Hiv/Aids, Stigma and Children. A Literature Review* (Cape Town: HSRC Press, 2007).

37 Link and Phelan, “Conceptualizing Stigma.” For a report discussing the relationship between stigmatisation and discrimination in the context of the HIV pandemic see: Aggleton., P, Wood., K and Malcolm., A, “HIV - Related Stigma, Discrimination and Human Rights Violations, A report by UNAIDS”. (English original, April 2005) ISBN 92 9 173,344 X Available at http://data.unaids.org/UNA-docs/JC999-HRViolations_en.pdf

38 D Reidpath and K Chan, “Hiv, Stigma, and Rates of Infection: A Rumour without Evidence,” *PloS Medicine* 3, no. 10 (2006).

39 Williams, Gonzalez-Medina, and Quan, “Infectious Diseases and Social Stigma.” In the great influenza pandemic of 1918/19 the poor were feared as carriers of the disease because they lived in cramped living conditions. The perceived increased incidence of disease within these communities increases the perceived risk of them being carriers of whatever infectious agent might be responsible for a recent outbreak of infectious disease. See: Barry, n.d., *The Great Influenza Story: The Epic Story of the Deadliest Plague in History* (New York: Viking).

40 A Courtwright, “Justice, Stigma, and the New Epidemiology of Health Disparities,” *Bioethics* 23, no. 2 (2009); “Stigmatization and Public Health Ethics.”

41 Guttman and Salmon, “Guilt, Fear, Stigma and Knowledge Gaps.”

42 For a good description of many such issues in various circumstances see Deliverable D1.6 “Human Rights, Stigmatization and Risk of Discrimination Against Specific Population Segments and Target Groups” from the project TELL ME - Transparent communication in Epidemics: Learning Lessons from experience, delivering effective Messages, providing Evidence. Project co-funded by the European Commission within the 7th Framework Programme. Grant No: 278,723. Available at: http://www.tellmeproject.eu/content/d16-human-rights-stigmatisation-and-risk-discrimination

43 For an lengthy (but not recent) discussion of such a definition see: F Foulke, “Definition and Nature of International Law,” *Columbia Law Review* 19, no. 6 (1919).

44 W Czapliński, “Jus Cogens and the Law of Treaties,” in *The Fundamental Rules of the International Legal Order*, ed. C Tomuschat and J Thouvenin (The Hague: Brill NV, 2006).

45 E Kuijt, *Humanitarian Assistance and State Soveriegnty in International Law* (Cambridge: Intersentia, 2015).

46 International Health Regulations (2005), WHA Res 58.3 23 May 2005 The new regulations were the fruit of ten years work attempting to redraft the old regulations in order to make them more suitable for modern needs. Recent instances of infectious disease outbreaks had indeed shown the previous version, adopted in 1959, to be out-dated. The new version was expanded considerably in scope and seeded more powers to the WHO in times of crisis. For more see: M Baker and D Fidler, “Global Health Surveillance under New International Health Regulations,” *Emerging Infectious Diseases* 12, no. 7 (2006).

47 L Gostin, “International Infectious Disease Law – Revision of the World Health Organisation’s International Health Regulations,” *Journal of the American Medical Association* 291, no. 21 (2004).

48 The IHR provisions require inter alia the application of the least intrusive and invasive medical examination that achieves the public health objective (Articles 17, 23, 31 and 43) and the need for prior express informed consent except in special circumstances (Article 23). States Parties must treat travellers undergoing health measures with respect for their dignity and human rights, and provide certain facilities to minimize their discomfort (Article 32).The Regulations also provide some protection as to confidentiality and lawful use of personal data collected under the IHR (Article 45) and introduce a general requirement of transparency and non-discrimination in the application of health measures (Article 42).

49 The framework was adopted at the World Conference on Disaster Reduction was held from 18 to 22 January 2005 in Kobe, Hyogo, Japan. This conference was organized by the United Nations Office for Disaster Risk Reduction. Since this text has been written the Hyogo framework has been replaced by its successor, the Sendai Framework agreed between 14th and 18th of March 2015. Whilst it brings with it innovations in a range of areas its requirements concerning communication remain largely the same.

50 P1

51 “Expected Outcomes”, p3

52 “Priorities for action 2005–2015”, p4.

53 “Priorities for Action” - Article 2 (d). Article (e) also demands that states “Establish, periodically review, and maintain information systems as part of early warning systems with a view to ensuring that rapid and coordinated action is taken in cases of alert/emergency.”

54 Importantly however, despite its potential relevance the Hyogo Framework is noticeable for its lack of any real enforcement mechanism. This means that individuals who may be effected by communications in a crisis have little way of actively enforcing the contents of the Hyogo agreement.The same is true of the Sendai framework – see foot note 59

55 A number of general rights of the ICCPR may be considered to have some relevance. Article 2(1) states that each State Party to the present Covenant undertakes to respect and to ensure to all individuals within its territory and subject to its jurisdiction the rights recognized in the present Covenant, without distinction of any kind, such as race, colour, sex, language, religion, political or other opinion, national or social origin, property, birth or other status. Article 26 of the ICCPR prohibits discrimination on any ground with regard to policy or law. Article 2(2) of the ICESCR declares: The States Parties to the present Covenant undertake to guarantee that the rights enunciated in the present Covenant will be exercised without discrimination of any kind as to race, colour, sex, language, religion, political or other opinion, national or social origin, property, birth or other status.

56 A Mehta and T Quinn, “Addressing Future Epidemics: Historical Human Rights Lessons from the Aids Pandemic,” *Pathological Immunity* 1, no. 1 (2016). Office), “A Human Rights Perspective into the Ebola Outbreak.”

57
*CCPR General Comment No. 6 The right to life (art.6) 04/30/1982*

58 the successful application of ‘the right to life’ in order to secure access to healthcare has been used sporadically in some jurisdictions. Concerning HIV is *Mendoza & Ors v Ministry of Public in Ecuador* Resn No 0749-2003-RA (28 Jan 2004) a court has for example held that the right to life encapsulates the right to health (see below). In that case it was stated that the right to life allowed citizens to take legal action in order to compel health authorities to enact appropriate laws to ensure that adequate resources are made available. In India, in the case, *Paschim Banag Khet Samity v State of West Bengal* (1996) 4 SCC 37 the right to life has been used to uphold a right to emergency medical treatment. In this case (of which analogous versions have occurred around the world an individual could not find a medical facility in his locality that was willing to admit him with the result that he was forced to seek treatment in a private institution.

59 The Right to Life is found within Article 2 of the European Convention on Human Rights

60 In Öneryildiz v Turkey for example the EtCHR stated that the state in question had a duty to protect individuals from methane gas explosions.

61 In Budayeva and others v Russia for example the EtCHR held that the state had an obligation to act to protect individuals from mud slides.

62 Following its revision, the 1996 revised European Social Charter, which came into force in 1999, is gradually replacing the initial 1961 treaty

63 It should be pointed out however that over 100 hundred nations have written an equivalent right to the ‘right to health’ into their national constitutions of legislation. See: E Kinney, “The International Human Right to Health: What Does This Mean for Our Nation and World?,” *Indiana Law Review* 34 (2001). This includes many developing nations. Chile for example has provided a constitutional right to health since 1925 See; E Kinney and B Clark, “Provisions for Health and Health Care in the Constitutions of the Countries of the World,” *Cornell International Law Journal* 37, no. 2 (2004).. In Argentina ‘the right to health’ was used by various community groups to obtain the provision of a vaccination against haemorrhagic fever. (See: Abramovich., V, “Argentina: The Right to Medicines’ in Litigating Economic, Social and Cultural Rights: Achievements, Challenges and Strategies” (Geneva: COHRE, 2003))

64 N Novogrodsky, “The Duty of Treatment: Human Rights and the Hiv/Aids Pandemic,” *Yale HumanRights and Decelopment Law Journal* 12 (2009).

65 Committee on Economic and Social Rights, General Comment No. 14

66 For more on this duty of progressive realisation see “Office of the United Nations High Commissioner for Human Rights – Factsheet 31 – The Right to Health”. Available at http://www.ohchr.org/Documents/Publications/Factsheet31.pdf

67 See for example The EU Commissions document ‘Strategy for Generic Preparedness Planning Technical guidance on generic preparedness planning for public health emergencies’ 2009 12 01

68 The enforcement of economic and social (ESC) rights has been relatively weak. See: M Ssenyonjo, *International Human Rights Law: Six Decades after the Udhr and Beyond* (Routledge, 2016). p88 Even with regards to civil and political rights many prominent international treaties are associated with weak enforcement mechanisms, especially in comparison with some regional agreements such as the ECHR. See: M Robbins, “Powerful States, Customary Law and the Erosion of Human Rights through Regional Enforcement,” *California Western International Law Journal* 35, no. 2 (2005).

69 Bayer, “Stigma and the Ethics of Public Health: Not Can We but Should We.”

70 *Kiyutin v Russia* No. 2700/10 10 March 2011 Para 53 Article 14 is designed in such a way that it is only activated when another convention right is engaged. This means that in order to use article 14, individuals must be presented with a situation that is ‘within the sphere’ of another right such as the freedom of private and family life or the right of liberty and security of person (article 8). For more discussion see: A Baker, “The Enjoyment of Rights and Freedoms: A New Conception of the ‘Ambit’ under Article 14 Echr,” *Modern Law Review* 69, no. 5 (2006). For a general review of article 14 and related case law see: R O′ Connell, “Cinderella Comes to the Ball: Art 14 and the Right to Non-Discrimination in the Echr,” *Legal Studies* 29, no. 2 (2009).

71 European Union Agency for Fundamental Rights, “Handbook on European Non-Discriminaiton Law,” (2010).

72 B Link and J Phelan, “Stigma and Its Public Health Implications,” *The Lancet* 367 (2006).

73 P Quinn., “The Problem of Stigmatising Expressions – the Limits of Anti-Discrimination Approaches.,” *International Journal of Discrimination and the Law* (Accepted, awaiting publicaiton) (2017c).

74 Quinn and De Hert, “Self Respect—a “Rawlsian Primary Good” Unprotected by the European Convention on Human Rights and Its Lack of a Coherent Approach to Stigmatization?.”

75 Quinn., “The Problem of Stigmatising Expressions – the Limits of Anti-Discrimination Approaches. .” In this paper the author uses the example of the anti-discrimination approaches adopted by the EU. Whilst they are capable of engaging certain expressive acts, they are only capable of doing so in specifically defined contexts. Such contexts do not include crisis communication. For more discussion of the directives see: G De Burca, “The Trajectories of European and American Antidiscrimination Law,” *Americal Journal of Comparative Law* (2011); T Simonovic Einwalter, “Far, but Not Far Enough: An Idealist Critique of the Racial Equality Directive,” *Croation Yearbook of European Policy & Law*, no. 4 (2008); K Henrard, “A First Substantive Ecj Judgement on the Racial Equality Directive: A Strong Message in a Conceptually Flawed and Responsivley Weak Bottle,” *Jean Monnet Working Paper* (2009).

76 One such example is Council Directive 2000/43/EC of 29 June 2000 implementing the principle of equal treatment between persons irrespective of racial or ethnic origin. This directive only applies to discirminaiton based on racial or ethnic origin.

77 Article 14 ECHR is an example of an example of an anti-discrimination approach that is not dependent upon an exhaustive list of categories because of the phrase “or other status”. This article has not however ever been applied to activity that is purely expressive in nature. See: P Quinn, “The Problem of Stigmatizing Expressions -the Limits of Anti-Discrimination Approaches,” *International Journal of Discrimination and the Law* 17, no. 1 (2017b).

78 The EU’s Data protection allows processing of data in the public interest without consent. Even where this is the case however important conditionality will apply to such processing, including the need to adhere to the normal provisions of data protection. See: “The Anonymisation of Research Data — a Pyric Victory for Privacy That Should Not Be Pushed Too Hard by the Eu Data Protection Framework?,” *European Journal of Health Law* 24 (2017a).

79 S Gutwirth et al., *European Data Protection: In Good Health?* (Springer, 2012).

80 Until May 2018 Directive Directive 95/46/EC on the protection of individuals with regard to the processing of personal data and on the free movement of such details in in force across Europe. Subsequent to this it will be the EU’s new General Data Protection Regulation (2016/679/EU). This later instrument aims at brining about harmonization across EU Member States.

81 Recital 26 of the GDPR for example confirms that it is not applicable where specific individuals can not be identified by the data in question.

82 Regulation (EU) 2016/679. Recital 26.

83 Other important initiatives relating to privacy are linked to human rights law, with the ECtHR’s case law under article 8 of the ECHR being one prominent example. Such case law has for example been important in delineating when public agencies (e.g. for security purposes) could use personal information without the consent of the individual involved. See for example the case of *Marper v UK. [2008] ECHR 1581*

84 C Harlow, “Global Administrative Law: The Quest for Principles and Values,” *The European Journal of International Law* 17, no. 1 (2006).

85 F Bignami, “Comparative Administrative Law,” in *The Cambridge Companion to Comparative Law*, ed. M Bussani and U Mattei (The George Washington University Law School, 2012).

86 S Rose-Ackerman and P Lindseth, “Comparative Administrative Law: An Introduction,” in *Comparaive Adminstrative Law*, ed. S Rose-Ackerman and P Lindseth (Elgar Publishing, 2011).

87 The sources of administrative law are many and varied. In the United States, the term ‘applied constitutional law’ is often used, reflecting the view that administrative law exists in order to make the state act within constitutional limits. See ibid. p1 For a discussion on this see: G Metzger, “Ordinary Administrative Law as Constitutional Common Law,” *Journal of the National Association of Administrative Law Judiciary* 30, no. 2 (2010). In France the *droit administratif* is provided primarily by a complex web of provisions produced by the *consiel d’etat* which in turn draws its authority from the constitution (See: G Bigot, *Introduction Historique Au Droit Administratif Depuis 1789* (Presses Universitaires de France (PUF), 2002). In the UK where there is no written constitution, the term administrative law has not historically been used but has gained traction in recent times (see: F Johnson Goodnow, *Comparative Administrative Law: An Analysis of the Administrative Systems, National and Local, of the United States, England, France and Germany* (London: Beard Books, 2000).) However there has for a long time existed legal doctrines that sought to ensure that parliamentary approval for ministerial actions existed and that such actions are made in accordance with certain principles, known as ‘natural law’, which are thought to be incumbent upon all decisions makers if they wish to operate in a just manner. In recent years the European Convention of Human Rights (the ECHR) has also provided an important source of administrative law in the UK and other European Jurisdictions. This has, to a certain extent, led to a convergence in administrative law across Europe (See: B Sordi, “Révolution, Rechsstaat and the Rule of Law: Historical Refections on the Emergence of Administrative Law in Europe,” in *Comparaive Adminstrative Law*, ed. S Rose-Ackerman and P Lindseth (Elgar Publishing, 2011).)

88 J Stewart, “Administrative Law in the Twenty-First Century,” *New York University Law Review* 78, no. 2 (2003).

89 Harlow, “Global Administrative Law: The Quest for Principles and Values.” p307

90 D Esty, “Good Governance at the Supranational Scale: Globalizing Administrative Law,” *Faculty Scholarship Series (Paper 428)* (2006b). p1524

91 P Craig, *Administrative Law (7th Ed)* (London: Sweet and Maxwell, 2012). The author discusses the rationale for procedural rights in chapter 12 p341

92 M Aronson, B Dyer, and M Groves, *Judicial Review of Administrative Action (3rd Edition)* (2004). P1. Quote taken from Harlow, “Global Administrative Law: The Quest for Principles and Values.” p193

93 See FN 42 concerning the EU “TELL ME” project.

94 D Esty, “Good Governance at the Supranational Scale: Globalizing Administrative Law,” *The Yale Law Journal* 115, no. 7 (2006a). See Part III

95 For a discussion on the evolution of this requirement in UK law see: M Eliot, “Has the Common Law Duty to Give Reasons Come of Age Yet?,” *University of Cambridge Faculty of Law Research Paper* (2012).

96 Guttman and Salmon, “Guilt, Fear, Stigma and Knowledge Gaps.”

97 A good example of such principles in the US context are the provisions in the Constitution guaranteeing freedom of speech and equal protection (i.e. non discrimination). These were introduced by the 1st and 14th Amendments respectively.

98 Quinn and De Hert, “Self Respect—a “Rawlsian Primary Good” Unprotected by the European Convention on Human Rights and Its Lack of a Coherent Approach to Stigmatization?.”

99 H Keller and A Stone Sweet, *A Europe of Rights: The Impact of the Echr on National Legal Systems* (Oxford: Oxford University Press, 2008). In the UK case of R (Daly) v Secretary of State for the Home Department the House of Lords stated for instance that administrators must justify themselves when they “limit” the convention rights of individuals.

100 As Craig, *Administrative Law (7th Ed)*.discusses on p21, the ECHR has transformed the process of judicial review at the heart of administrative law in the UK. Courts must now analyse administrative decisions in the light of ECHR principles and where national legislation is incompatible they must, under the Human Rights Act (1998) issue a declaration of non-conformity.

101 Such a focus is demonstrated for example by the notion of ‘formal’ and ‘informal rule making’ and ‘adjudications’ in the American Admisitrative Procedure Act and the concepts of *acte administratif unlilateral* and *ordonnancement juridique* in the French *droit administratif.* Similarly illustrative examples can be found in other civil law systems of administrative law such as the German *Verwaltungsverfahrensgesetz* and can be easily discerned in the practice of common law systems such as the UK.

102 In UK law there is a presumption that “Generally, a person will not be acting in the course of his official duties as a servant of the Crown when doing something that is prohibited by the general law.” This quote is taken from a pamphlet prepared for Members of the Office of the UK Parliamentary Council. Available at https://www.gov.uk/government/uploads/system/uploads/attachment_data/file/493665/Crown_Application.pdf. For more see: Craies on Legislation (10th ed., 2012) 11.5.6 to 11.5.7 and 11.5.16 to 11.5.21. A similar situation exists in most common law and civil law states. In New Zealand for example. The US in comparison recognises a greater range of immunities for public employees. Whilst no general system of immunity from criminal prosecution for public employees exists, immunity may be available under certain circumstances however. See: Larkin, 2013, “Sauce for the Goose Is Sauce for the Gander”: “Treating Private Parties and Government Officials Alike under the Criminal Law,” *Legal Merorandum*.

103 J Morgon, “Us Hate Crime Legislation: A Legal Model to Avoid in Australia,” *Journal of Sociology* 38, no. 1 (2002).

104 In the UK for example such provisions are contained within the Public Order Act of 1986.

105 Belgian criminal law (in article 453 of the criminal code), for example recognises offences of incitation to both ‘hatred’ and ‘discrimination’. The groups protected by such laws are numerous and of broad application. They are ‘racial origin’, ‘skin colour’, ‘family ascendance’, ‘national or ethnic origin’, ‘present nationality’,‘gender’, ‘sexual orientation’, ‘civil state’, ‘birth’, ‘age’, ‘fortune’, ‘religious or philosophical convictions’, ‘health status (present or future)’, ‘disability’, ‘language’ ‘membership of a union’, ‘physical characteristics’, ‘genetic characteristics’ or ‘social origin.

106 There are some exceptions where sates possess wide ranging hate speech laws that are for example capable of protecting undefined groups. Such legislation is however relatively rare with Germany being one of the most important.. For more on the German law see: W Brugger, “The Treatment of Hate Speech in German Constitutional Law (Part I),” *German Law Journal* 3 (2002a); “The Treatment of Hate Speech in German Constitutional Law (Part Ii),” *German Law Journal* 4, no. 1 (2002b).

107 S Lewis et al., ““I Don’t Eat a Hamburger and Large Chips Every Day!” A Qualitative Study of the Impact of Public Health Messages About Obesity in Obese Adults,” *BMC Public Health* 10 (2010).

108 See UK Public Order Act (1986).

109 See the Article 24 of the ‘loi du 29 juillet 1881’in France for example.

110 For further discussion on a range of hate speech law in several European countries see: M Van Noorloos, *Hate Speech Revisted - a Comparative and Historical Perspective on Hate Speech Law in the Netherlands and England and Wales* (Cambridge: Interstentia, 2011); E Janssen, *Faith in Public Debate. On Freedom of Expression, Hate Speech and Religion in France & the Netherlands* (Cambridge: Interstentia, 2015).

111 Guttman and Salmon, “Guilt, Fear, Stigma and Knowledge Gaps.”

112 Intervention by Jean-Marc Sauvé (vice president du Consiel d’etat) “Quelle déontologie pour les hauts fonctionnaires?” Presentation made at the Ecole Nationale dÁdministraiton on the 27th March 2013. Available at: http://www.conseil-etat.fr/Actualites/Discours-Interventions/Quelle-deontologie-pour-les-hauts-fonctionnaires (In French)

113 A De Becker, “Juridische Status Van Een Ethische Code: Waar Plaats Je Zo’n Code in De Hiërarchie Van De Continentale Normen?,” *Bestuurskunde* 23, no. 4 (2014). (In Dutch)

114 The current UK Civil Service code of conduct (dating from March 2015) is available at https://www.gov.uk/government/publications/civil-service-code/the-civil-service-code

115 See for example the the “Deontogishe Code Vlaamse Overhied (applicable in the Flemish Region) which demands civil servant to act objectivity and to avoid discrimination”. Avaialble at https://overheid.vlaanderen.be/deontologische-code (In Dutch)

116 The French law of conduct for civil servants demands inter alia that public servants show neutrality when communicating in public with a view to maintain the impartiality of the state. See: J Auby,*, La Fonction Publique* (Paris: Dalloz, 2002). p300 (In French)

117 See for example for the Code of Conduct of for Federal Communicators (Code de déontologie des communicateurs fédéraux) Available at: http://www.fedweb.belgium.be/sites/default/files/downloads/broch_commcollection10_deontologischecode_fr.pdf (In French)

118 De Becker, “Juridische Status Van Een Ethische Code: Waar Plaats Je Zo’n Code in De Hiërarchie Van De Continentale Normen?.”

119 J Maesschalck and F Schram, “Meer Dan Een Brochure of Affiche: De Deontologische Code Als Kernelement Van Een Effectief Ambtelijk Integriteitsbeleid,” *Burger, Bestuur & Beleid* 3, no. 1 (2006). (In Dutch)

120
http://www.gmc-uk.org/Good_medical_practice___English_1215.pdf_51527435.pdf

121 In Belgium the ‘Ordre des médecins’ is the relevant body. Its ethical code (Code de déontologie médicale) can be found at https://ordomedic.be/fr/code/contenu/ (In French)

122 In France the ‘Le Conseil mational de l’Ordre des médecins’ is the primary professional body. Its main ethical code **(**Code de déontologie médicale**)** can be found at https://www.conseil-national.medecin.fr/sites/default/files/codedeont.pdf (in French)

123 The German Medical Association (*Bundesärztekammer (BAK)) is unlike the other examples presented here in that it is not a unitary organisaiton but is rather a composite of organizations rom each German Lander. For a model code see:* *http://www.bundesaerztekammer.de/fileadmin/user_upload/downloads/pdf-Ordner/MBO/MBO_EN_Novellierung_2015.pdf*

124 The ethical code produced by the Irish Medcial Council can be found at http://www.medicalcouncil.ie/News-and-Publications/Reports/Guide-to-Professional-Conduct-Ethics-8th-Edition.html

125 J Jowell and M Oliver, *The Changing Consitution* (Oxford: Oxford University Press, 2004). p384 In the UK for example this has been an accepted principle of the UK constitutional arrangement since the sixteenth century. Whilst UK courts have been willing to intervention the supply side of government expense i.e. taxation they have been extremely reluctant to get involved in matters of how money is spent. Even in the second half of the twentieth century where judicial intervention increased in areas such as welfare payments, intervention has often been limited to procedural questions querying the application for example of principals of natural justice to individual cases of dispute. For more on natural justice see: Craig, *Administrative Law (7th Ed)*.chapter 12

126 Hood and Margretts, *The Tools of Government in the Digital Age*. See for example (in chapter 4) Hood and Margretts’ description of the concept of ‘treasure’. This concept reflects that fact that it is through the allocation of funds that they state is able to control the activities of its various departments and sub organisations.

127 Morgan and Yeung, *An Introduction to Regulation*.. See chapers on ‘dominium’ and ‘imperium’

128 In the well known case of R. v. Secretary of State for Foreign Affairs, ex part World Development Movement [1995] 1 All ER 617, the Court intervened in the case of a UK government grant to build a dam in Peru. Even on that occasion however where the court questioned the spending as inappropriate the UK Government was able to go ahead with funding the grant by deploying funds from another source.

129 See the comments by Craig, *Administrative Law (7th Ed)*. discussed in the previous section. See also section 6 of this document where the position of administrative law viz*-á-*viz expressive activity is discussed.

130 For a comparison of the makeup and role of the *Cour des Comptes* with similar institutions in other European countries see: S Jacob, *Institutionnaliser L’évaluation Des Politiques Publiques: Étude Comparée Des Dispositifs Institutionnels En Belgique, En France, En Suisse Et Aux Pays-Bas* (Peter Lang, 2005). (In French)

131 This competence is outlined in Article 47-2 of the French constitution. The role of the Cour des Comptes was recently updated by loi consitituntionelle du 23 juillet 2008.

132 Article 47 de la Constitution de la Cinquième République française

133 Loi organique n°2001-692 du 1er août 2001 relative aux lois de finances provides that public servants must show effectiveness (effecacité) in the spending of credits that have been attributed to them.

134 Code des jurisdictions financiérers, Article R 143-3

135 J Von Hagen, “Fiscal Rules, Fiscal Institutions, and Fiscal Performance,” *The Economic and Social Review* 33, no. 3 (2002).

136 Figures for tax year 2015/2016. See: http://www.nhs.uk/NHSEngland/thenhs/about/Pages/overview.aspx

137 The NHS as an organisation is necessarily categorised as having a high degree of decentralised budgetary control. See: N Flynn, *Public Sector Management* (SAGE, 2007).

138 See C Hood, “Intellectual Obsolescence and Intellectual Makeovers: Reflections on the Tools of Government after Two Decades,” 20, no. 127-144 (2007). Chapter 2

139 See also chapter 2 of Hood and Margretts, *The Tools of Government in the Digital Age*.

140 For a discussion in the context of the recent outbreak of Ebola in west Africa see: Böl, “Risk Communication in Times of Crisis - Pitfalls and Challenges in Ensuring Preparedness Instead of Hysterics.”
